# Circadian clock: a regulator of the immunity in cancer

**DOI:** 10.1186/s12964-021-00721-2

**Published:** 2021-03-22

**Authors:** Zhen Zhang, Puhua Zeng, Wenhui Gao, Qing Zhou, Ting Feng, Xuefei Tian

**Affiliations:** 1 Department of Internal Medicine, College of Integrated Chinese and Western Medicine of Hunan University of Chinese Medicine, 300 Xueshi Road, Changsha, 410007 Hunan People’s Republic of China; 2 Hunan Key Laboratory of TCM Prescription and Syndromes Translational Medicine, Hunan University of Chinese Medicine, Changsha, 410208 People’s Republic of China; 3 Affiliated Hospital of Hunan Academy of Traditional Chinese Medicine, Changsha, 410006 People’s Republic of China; 4 Department of Andrology, The First Affiliated Hospital of Hunan University of Chinese Medicine, Changsha, 410208 People’s Republic of China

**Keywords:** Circadian clock, Cancer, Cancer-immunity cycle, Chrono-immunotherapies, Tumor microenvironment (TME)

## Abstract

The circadian clock is an endogenous timekeeper system that controls and optimizes biological processes, which are consistent with a master circadian clock and peripheral clocks and are controlled by various genes. Notably, the disruption of circadian clock genes has been identified to affect a wide range of ailments, including cancers. The cancer-immunity cycle is composed of seven major steps, namely cancer cell antigen release and presentation, priming and activation of effector immunity cells, trafficking, and infiltration of immunity to tumors, and elimination of cancer cells. Existing evidence indicates that the circadian clock functions as a gate that govern many aspects of the cancer-immunity cycle. In this review, we highlight the importance of the circadian clock during tumorigenesis, and discuss the potential role of the circadian clock in the cancer-immunity cycle. A comprehensive understanding of the regulatory function of the circadian clock in the cancer-immunity cycle holds promise in developing new strategies for the treatment of cancer.

**Video Abstract**

**Supplementary Information:**

The online version contains supplementary material available at 10.1186/s12964-021-00721-2.

## Background

According to an assessment of the World Health Organization (WHO) in 2015, cancer is the major cause of death and the most significant barrier to the lengthening of lifespan expectancy. The incidence and mortality of cancer have sharply increased in every country in the twenty-first century [1]. At present, the first-choice treatment for various neoplasms is surgical excision and occasionally in combination with other therapies, including chemotherapy, radiotherapy, and targeting therapies. However, these treatments effect are not satisfactory [2]. Over the last few years, immunotherapy has become the breakthrough treatment with mild side effect and survival rates in the most malignant tumors.

Circadian rhythms are dominated by an endogenous time-keeping system in mammals such that synchronization with the 24-h environmental cycle generated by the Earth’s rotation is achieved. Existing evidence indicates that approximately 10% of the human genome is controlled by the circadian clock, which also affects a variety of physiological processes, such as sleep–wake cycles, body temperature cycles, digestive and cardiovascular processes, endocrine systems and immunity systems [3–6]. In recent years, the effects of the circadian clock on tumor immunity have been studied; however, the role of the circadian clock in tumor immunity remain unclear. Here, we review the mechanism that places tumor immunity under the control of the circadian clock; this mechanism may be applied in developing biological clock targets and chrono-immunotherapies for the treatment of tumors.

## Tumor microenvironment and immune system

The tumor microenvironment (TME) is composed of tumor mass surrounded by fibroblasts, stromal cells, immune and inflammatory cells, and the extracellular matrix (ECM) [7]. In the TME, immune cells dominantly exert effects impact over tumor growth, immune surveillance, tolerance, and escape. Moreover, multiple immune cells reportedly participate in the anti-tumor process, including natural killer (NK) cells, tumor-associated macrophages (TAMs), neutrophils natural killer T (NKT) cells, dendritic cells (DCs), T- lymphocytes, and B-lymphocytes [8–10].

An increasing number of evidence strongly support that the immune conditions of patients can be used to the predict outcomes of a broad range of solid tumors, such as non-small-rcell lung cancer (NSCLC), liver cancer, and breast cancer [11–14]. The general consensus revealed that high macrophage infiltration in TME indicates resistance to therapy and poor prognosis [15]. Several early clinical trials targeting macrophages have been successfully performed [16]. The activation of a broad spectrum of T cells was suppressed in the immunosuppressive TME, which eventually facilitated the circumvention of the immune system and the escape of malignant cells, thereby promoting cancer progression and metastasis [17]. The number of CD4+ T cells have been significantly associated with prognosis of NSCLC[18]. Thus, the inhibiting expression of CD4 + T cells is a potential approach for lung cancer immunotherapy [19]. Apart from immunity cells, a serious of chemokines or chemokine receptors regulate the migration of different subsets of lymphocyte into TME and contribute to cancer progression and therapeutic outcomes [20]. Negative regulators of immune activation, including cytotoxic T-lymphocyte-associated antigen 4 (CTLA-4), programmed death receptor1 (PD-1), can succeed in a struggle against lymphocyte exhaustion in the tumor microenvironment, thus triggering the antitumoral function of lymphocyte cells [21, 22].

**Fig. 1:**
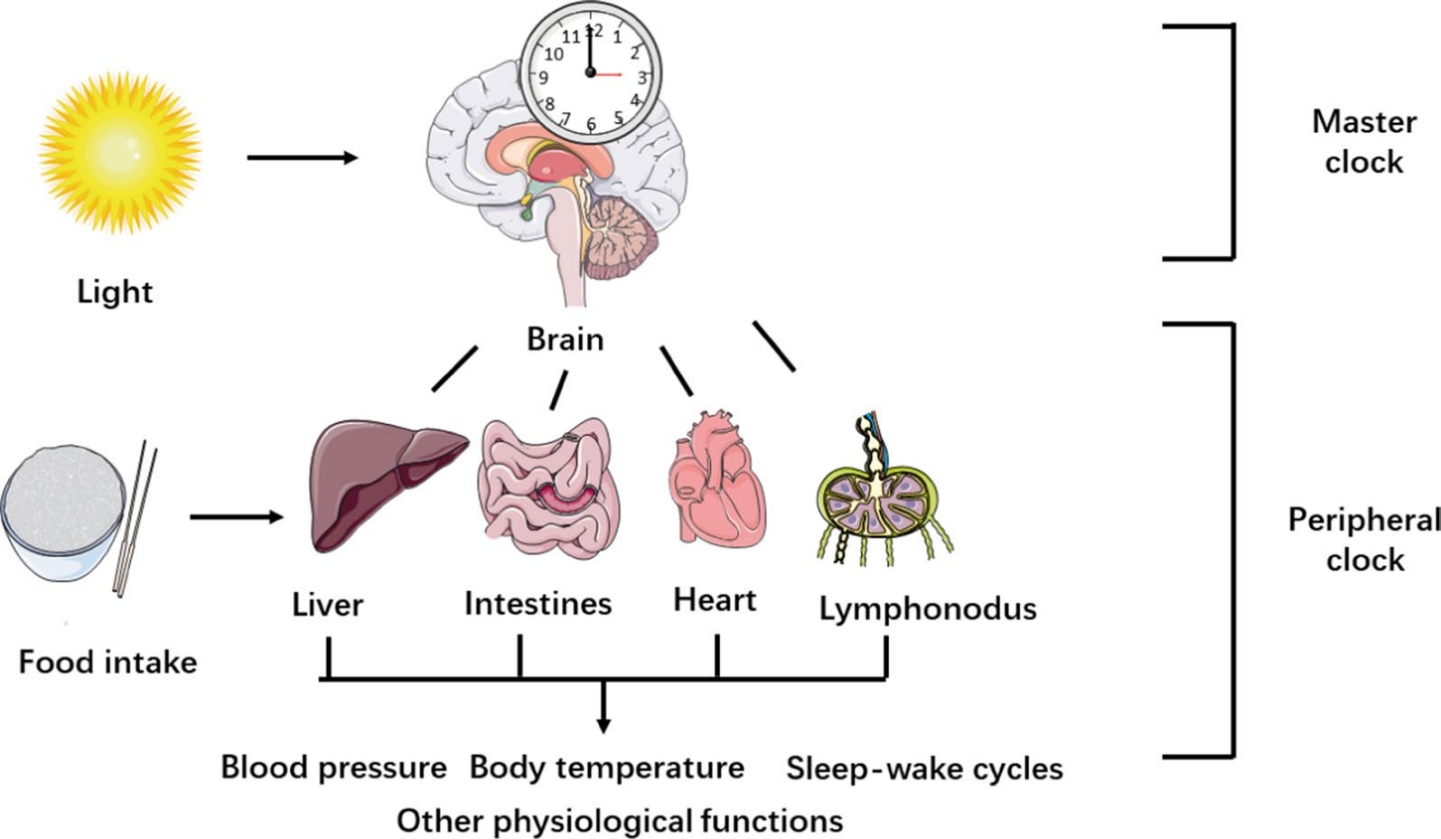
The master clock and peripheral clocks. Light provides entrainment signals for the master clock, whereas food intake mainly stimulates peripheral clocks. The master clock and peripheral clocks coordinate in regulating many biological processes in the human body

**Fig. 2:**
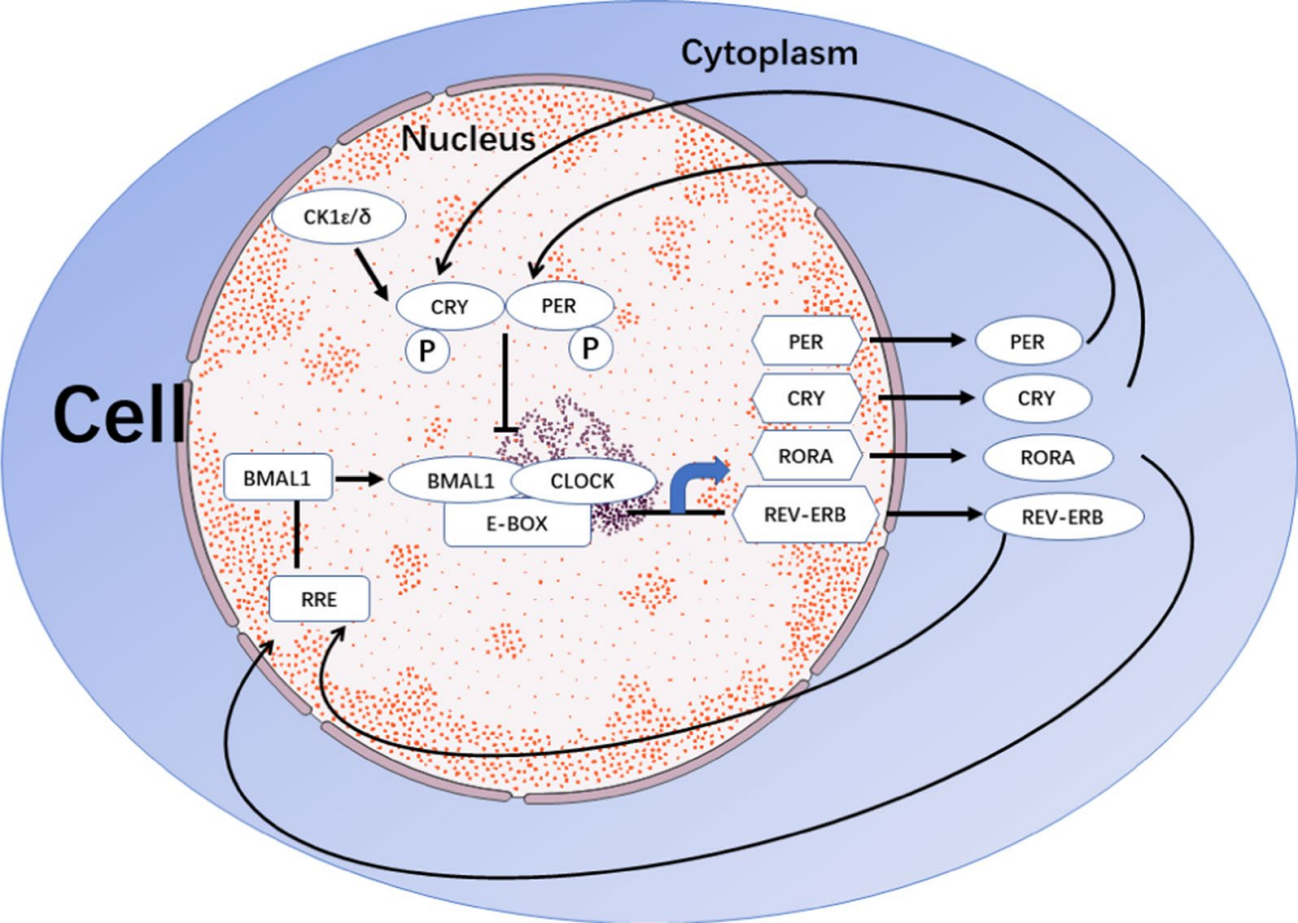
The protein clock BMAL1 heterodimerizes with CLOCK to initiate the transcription of targets genes through E-boxes. PER and CRY proteins inhibit the expression of BMAL1 and CLOCK, respectively, whereas RVE-ERBα/β does the opposite by binding to RREs. Additionally, CRY and PER are also negatively regulated by CK1ε/δ via phosphorylation

The mechanism of tumor immunity is complex and a series of self-sustaining steps must be geared to be processed and expanded to kill cancer cells effectively [23]. Effective anti-tumor immunity can be established through a circulation mechanism, that is the cancer-immunity cycle. The cancer-immunity cycle is composed of seven major steps, namely cancer cell antigen release and presentation, priming and activation of effector immunity cells, trafficking, and infiltration of immunity to tumors, and elimination of cancer cells. In the first step, antigen-presenting cells (APCs) can effectively acquire and process neoantigens from dying cells, including targeting antigens for cross-presentation and tumor cells. In order to yield T cell immune response against tumors, it must be accompanied by distinct cytokine release and signal activation such that peripheral tolerance to the tumor antigens is induced. Then, DCs present tumor-associated antigens (TAAs) to T cells. Subsequently, effector T cells are primed and activated to eliminate cancer-specific antigens. Under chemokines release, the activated effector lymphocytes traffic to and infiltrate tumor tissues. Subsequently, cytotoxic T-lymphocytes (CTLs) distinctly recognize and combine with malignant cells. Finally, cancer cells are killed, and additional antigens are released, thereby tumor immune response in succeeding cycles. Given the potential feasibility of cancer-immunity cycle oscillation, it is critical to understand how the circadian clock modulates immunity (Figs. 1, 2).

## Genes and molecular mechanism of the circadian clock in mammals

**Fig. 3:**
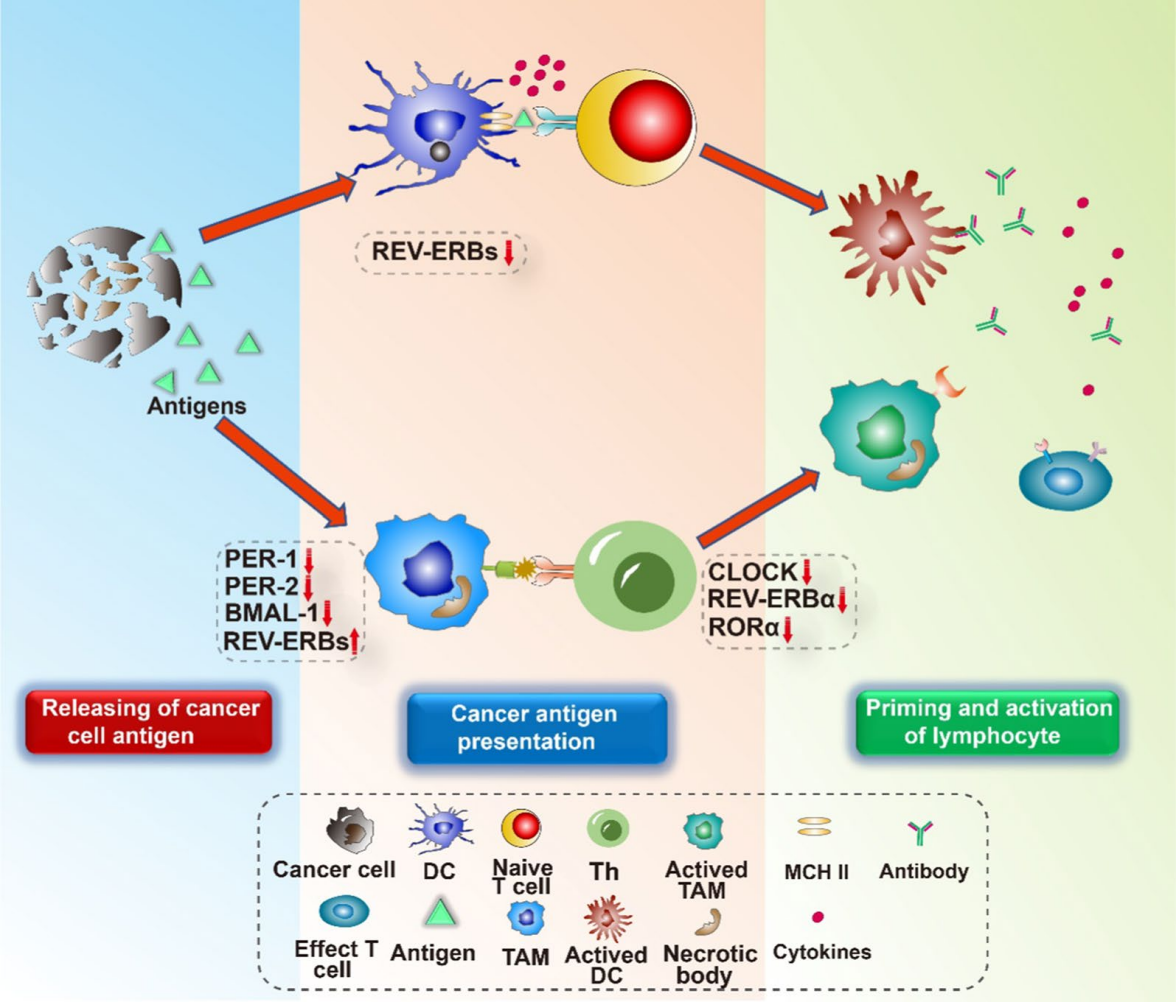
Initiate anti-cancer immunity: releasing of cancer cell antigen, cancer antigen presentation, and priming and activation. Tumor cell death is accompanied by antigen release. Dendritic cells present antigens to naïve T cells. Then, the naïve T cells prime and activate the effector T cells. On the other hand, the antigen can also be presented by TAMs. Additionally, TAMs stimulate Th cells to produce cytokines. The circadian clock components take part in these processes

**Fig. 4:**
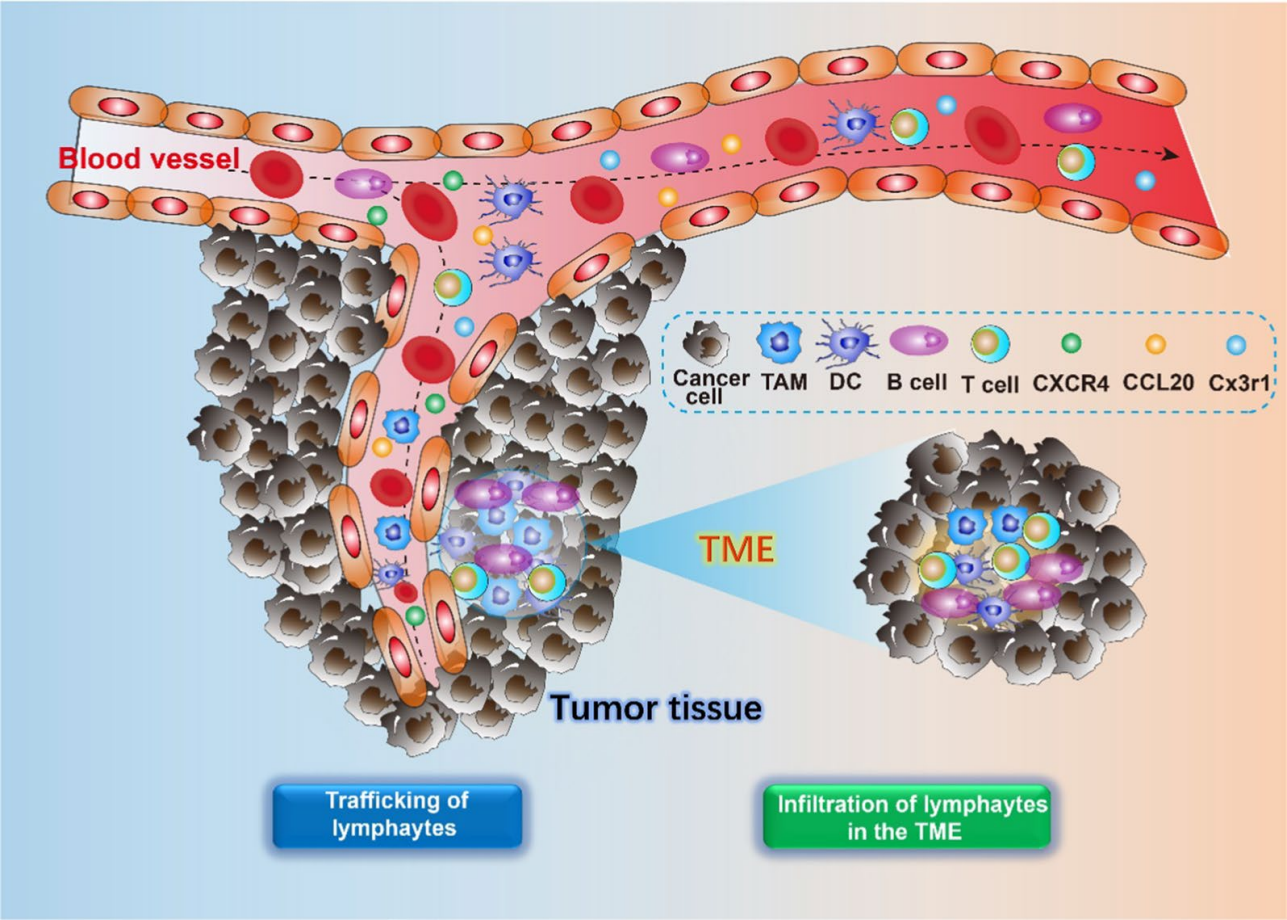
The processes of lymphocyte trafficking and infiltration in cancer. The chemokines, including CXCR4, CCL20, Cx3r1, modulates the lymphocytes trafficking and infiltration into the TME

In mammals, the circadian system is an integral regulatory system composed of a master clock and peripheral clocks. The master clock exists in the suprachiasmatic nucleus (SCN) of the brain, whereas the peripheral clocks reside in peripheral tissues and organs, including the liver, skin, lungs, and kidneys. The fact that the circadian clock resides in nearly every mammalian cell has indicated modulatory complexity. When mammals are exposed to light, the retina is activated and transmits information to the SCN. The SCN receives, converts, and integrates the information from the external environment to tissues, which synchronizes the signals to regulate its own rhythms and peripheral rhythms and maintain robust circadian oscillations in neuronal activity [24]. The clocks can also be reset in response to other external signals, including food intake and hormones, independent of the master clock [25, 26]. Conflict between the SCN and locally derived signals may lead to the malfunction of the circadian clock, and the impairment of temporal control of cell-specific programs (Figs. 3, 4).

**Fig. 5:**
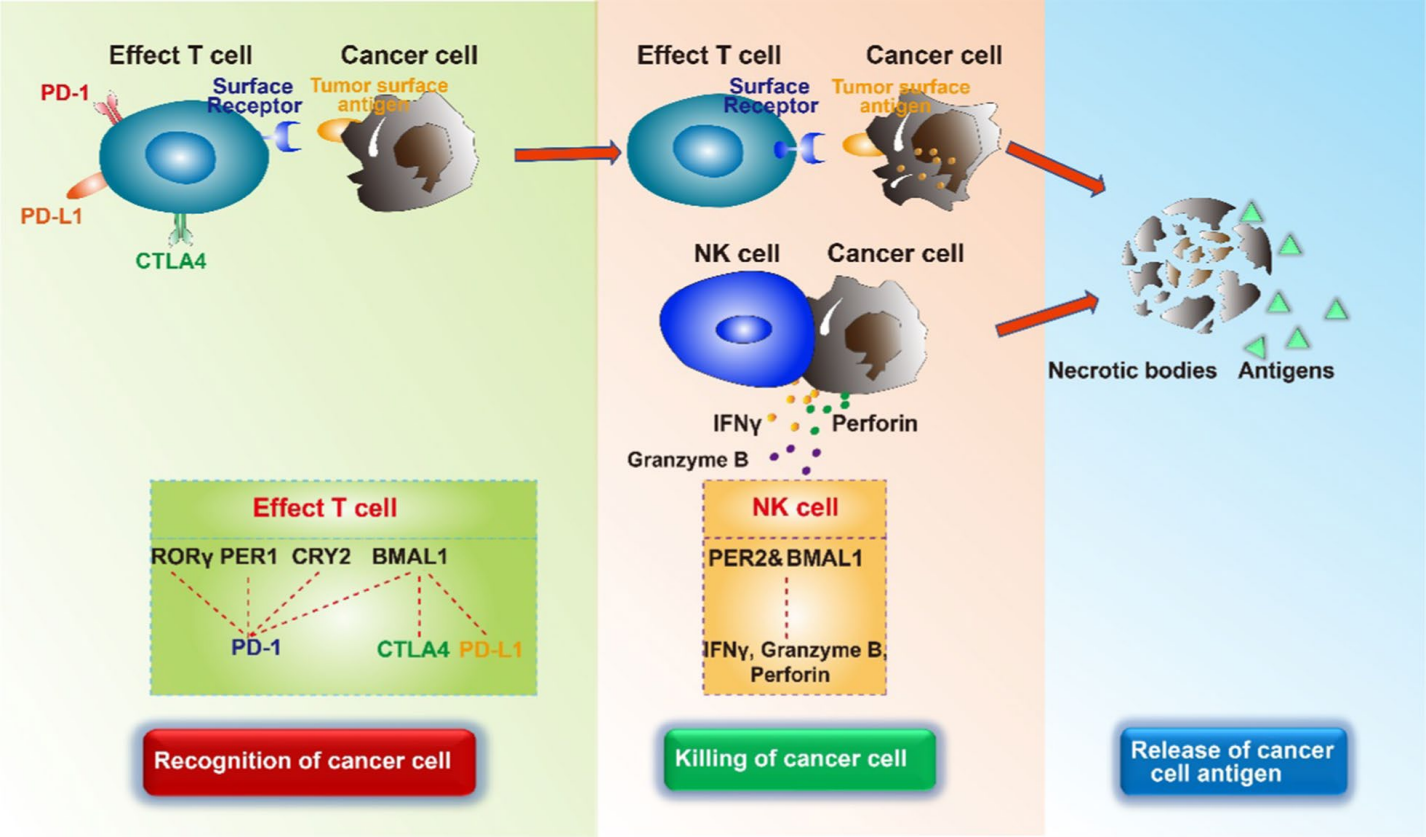
The processes of recognition of cancer cell, killing of cancer cell, and releasing of cancer cell antigens. T cells recognize tumor cells through surface receptors; the activation of NK cells in tumor sites directly destroys malignant cells without prior sensitization. The circadian clock, including RORγ, PER1, CRY2, and BMAL1 negatively regulate the expression of PD-1 in effector T cell. BMAL1 also negatively regulates the expression of CTLA4 and PD-L1 in effect T cells. PER1 and BMAL1 can increase the secretion of IFN-γ, granzyme B, and perforin in NK cells. When cancer cells are killed, cancer cell antigens and necrotic bodies are released in blood

At the cellular and molecular levels, the circadian rhythms that emerge from the master and peripheral clocks are almost similar. The core molecular clock is comprised of two main autoregulatory interlocking transcription-translation feedback loops (ITTFs), which counter-modulate each other to produce a circadian cycle of gene expression, and exists in the SCN and peripheral tissues [27, 28]. At least 14 core and 37 related circadian clock genes participate in the process of circadian clock [29–32]. Note that circadian locomotor output cycles kaput (*Clock*) and brain and muscle Arnt-like protein-1 (*Bmal1*) are regarded as activators of the circadian clock, whereas PERIOD (PER-1; PER-2; PER-3) and cryptochrome (CRY-1; CRY-2) proteins are regarded as inhibitors. In the main loop, BMAL1 and CLOCK, which can be replaced by its paralog neuronal PAS domain protein 2 (NPAS2), form a heterodimer that bind to enhancer element of E-box [33] to activate the transcription of target genes and modulatory proteins, including PER-1, PER-2, PER-3, CRY-1, and CRY-2 [34, 35], thereby forming a positive feedback loop. However, other studies have reported that PER-1, PER-2, CRY-1, and CRY-2 play essential roles in the regulation of circadian clock, whereas PER-3 does not have any circadian phenotype [36–39]; the reason for this remains unclear. In the second major loop, the translated proteins of PER and CRY families in the cytoplasm form hetero-multimeric complexes and are transported to the nucleus, which negatively regulates the activation of CLOCK/BMAL1 (NPAS2) heterodimers [40]. These positive and negative ITTFs circulate with approximately 24-h circadian periodicity. In the above process, casein kinase 1 (CK1) ε/δ also restricts negative feedback potential of PER and CRY via degeneration or phosphorylation [41–43]. Apart from the above ITTFs, the heterodimer CLOCK/BMAL1 drives the rhythmic transcription of nuclear receptor subfamilies (REV-ERBs), and retinoid-related orphan receptors (RORs) by binding to the E-boxes in their promotional genes. RVE-ERBα/β and RORα/β can regulate the transcription of *Bmal1*. RORα/β promotes the expression of *Bmal1* by binding to ROR respective elements (RRE), and thereby forms a positive feedback loop, whereas RVE-ERBα/β do the opposite [44, 45]. These two feedback loops are the basic building components of the cellular clock. However, many more genes are directly or indirectly involved in the clock machinery, resulting in rhythmic expression of clock-controlled genes via E-boxes, D-boxes, and RREs. Mutation in these genes results in the malfunction of behavior and physiology, and in the alteration in the period, phase, or amplitude of circadian rhythms. As a result, these molecular participate in various ailments, including cancer [46–48] (Fig. 5).

## Disruption of the circadian clock contributes to cancer

Over the past years, studies have shown that disruption of the circadian rhythm contributes to the incidence and development of various cancer [49, 50]. Previous studies have revealed that shift work is implicated in tumorigenesis [51, 52]. Women who work at night instead of days exhibit an approximately 10% increased risk of breast cancer [53–55]. In another study, intermittent/periodic fasting and fasting-mimicking diets, reportedly can cause organic fat weakening without body mass change, speed up immune system renewal, increase the risk of cancer [56]. Of note, the disruption of life cycle oscillation causes the increase of spontaneous cancer in chronic jet-lag mouse model. For example, Minami et al. discussed that chronic jet-lag mice showed the short lifespan, splenomegaly, and the accelerated development of liver cancer [57]. In addition, the chrono-disruption of the circadian clock is crucial in metabolic and immunologic changes and is implicated in non-alcoholic fatty liver disease/nonalcoholic steato-hepatitis/hepatocellular cancer [58].

In studies wherein circadian clock gene-mutant animal models were used the disruption of circadian clock components, including BMAL1, PER subfamily, CRY-1, CRY-2, and so on, implicate enhanced oncogenesis. Compared to adjacent and benign tissues, tumor tissues from pancreatic ductal adenocarcinoma patients expressed significantly lower levels of circadian clock, including PER-1, PER-2, PER-3, CRY-2, and CK1ε, which are related to poor prognosis [59]. *Bmal1* is a key component in hematologic malignancies, and the inactivation of BMAL1 promotes the progression of hematologic malignancies by disrupting the cellular circadian rhythm and impairing the characteristic circadian clock expression pattern of genes, including C-MYC, catalase, and p300 [60]. Additionally, altered circadian rhythms have been reported to be correlated with the prognosis of breast cancer [61].The exist circadian rhythm and the core circadian gene *Bmal1*, persist in malignancy breast cancer cells [61]. Tumor hypoxia-induced acidosis decreases the transcription activation and protein stability of BMAL1 to promote breast cancer metastasis in vitro [62]. Moreover, members of PER subfamily, including PER-1, PER-2, and PER-3, have been reported to be lowly expressed and play significant role in NSCLC patients [63]. Furthermore, the low expression of PER-2 is reportedly negatively linked to poor differentiation, big tumor sizes, high TNM stage, and lymph node metastasis in NSCLC patients; the overexpression of PER-2 inhibits the migration and invasion of NSCLC cell lines [63, 64]. In an analysis of the differential expression of the circadian genes in hepatocellular carcinoma and paired noncancerous tissues, the expression levels of some circadian genes, including PER-1, PER-2, PER-3, CRY-2, and TIM, were decreased; this was reportedly a result of promoter methylation, the overexpression of EZH2, or of other factors rather than of genetic mutations [50]. These studies indicate that the circadian clock gene can regulate the occurrence and development of tumors.

## Oscillation parameters of immunity

The number of circulating lymphocytes and cytokines in the blood is the key parameter for the status of the immune system of the human body. Lymphocytes in the TME possess intrinsic clocks and have been linked to antitumor response and poor patient’s outcomes [65, 66]. Generally, the count of hematopoietic stem progenitor cells (HSPCs), and most mature leukocytes are displayed regularly in numerous frequencies [67]. According to the activity-rest phase of species, the immune system shows high-amplitude circadian rhythms in both count and function measures, including NK cells, monocytes, DCs, B cells, and T cells in mammals [68–70]. For example, the number of undifferentiated T cells and NK cells exhibits the expected pronounced circadian rhythm with an acro-phase during sleep phase and nadir during activity phase in human blood [71, 72]. Further analysis indicated that the number of naive CD8+ and CD4+ T cells peaks during the rest phase, and the effector CD8+ T cells during the active phase [71]. In addition to lymphocytes, anti-inflammatory cytokines, including interleukin-10 (IL-10) and IL-4, are also present in an oscillating manner, and are induced during the onset of the active phase in the human body [73]. In contrast, the basal plasma levels of pro-inflammatory cytokines in human, including, tumor necrosis factor-α (TNF-α) and IL-1β, are generally high during the rest phase [74]. Possibly, these observations may be associated with the brain’s major stress-hormone systems, that is, by release of glucocorticoids and catecholamines [71].

However, compared with those of healthy subjects, some parameters of biologic rhythms in the immune system are disordered in cancer patients. Gianluigi et al. reported that the lymphocyte subsets, including total T cells and total B cells exhibit a circadian oscillation with nocturnal acrophase, whereas T cytotoxic cells exhibit circadian oscillation with diurnal acrophase in heathy participants [75, 76]. However, in patients with lung cancer, except for T helper (Th) cells, all lymphocyte subsets reportedly loss circadian oscillation [75, 76]. Abnormal proportions and nocturnal variations of different lymphocyte subpopulations may alter the immune function of patients with NSCLC and destroy the interaction between accessory cells and lymphocyte subsets [77]. These observations may have implications if increasing the effect of tumor immunotherapy by enhancing the rhythmicity of tumor cell parameters is feasible.

## Circadian clock regulates tumor immunity cycle

The circadian clock functions as a gate that controls many aspects of immune functions in relation to cancer, including cancer cell antigen release and presentation, priming and activation of effector immunity cells, trafficking, and infiltration of immunity to tumors, and elimination of cancer cells [78].

### Circadian clock components initiate antigen presentation and T cell priming and activation

Tumor cells usually contain TAAs and tumor-specific antigens (TSAs). These antigens are released to the plasma during tumor cell death and are then captured by DCs and macrophages [16]. DCs, macrophages, and B cells are potent antigen-presenting cells (APCs) in the human body. When stimulated by antigens or inflammatory cytokines, including IL-1β, TNF-α, TGF-β, immature DCs can differentiate into mature DCs and express MHC II molecules on their surface. Simultaneously, the expression levels of costimulatory molecules and adhesion molecules are also significantly increased on their surface. Then, antigens are extracted from peripheral tissues to prime and activate T cells. In addition, DCs can secrete IL-12, which makes T cells in TMEs differentiate into T helper (Th) cells to promote the elimination and growth inhibition of tumor cells [79].

Functional molecular clocks in DCs such as CLOCK, PER, and BMAL-1 show daily oscillations and indicate that the host DCs are under circadian regulation [80]. Moreover, the expression of pro-inflammatory cytokines, co-stimulatory molecules, and MHC II in DCs are increased in *Rev-Erbα-* and *Rev-Erbβ-*deficient mice [81]. This indicates that *Rev-Erbs* negatively modulate development and activation of DCs and are important in antigen presentation [81]. These studies provide us with clues that, to some extent, clock genes can regulate the function of DCs; however, the extent of their impact and related mechanisms need to be further explored.

Macrophages, also known as TAMs, are one of the most abundant components of the TME. TAMs display a broad spectrum of activation states with distinctive phenotypes and functions [82]. In this broad spectrum of activation states, TAMs are categorized as two polarized extremes, namely, the M1-like TAMs (or classically activated, pro-inflammatory/anti-tumoral) macrophages and the M2-like TAMs (or alternatively activated, anti-inflammatory/pro-tumoral) [83]. M1-like TAMs, as central regulators of the complex TME, exert integrated effects to promote the recruitment and activation of T helper (Th) cells via the secretion a variety of cytokines [84, 85]. Furthermore, M1-like TAMs are involved in pathogen-associated molecular patterns (PAMPA), induce the maturation of APCs, and directly phagocytose cancer cells [86]. Macrophages have functional molecular clocks, as evidenced by the daily circadian oscillations in clock gene expression [80]. In a study, an M1-like pro-inflammatory phenotype was observed in macrophages isolated from mice with relatively disrupted *Per1* and *Per2* clock genes [87]. Moreover, the complete deletion of *Bmal1* in macrophage cells has been reported to directly result in a decrease production of the master antioxidant transcription factor NRF2, which diurnally modulates ROS in myeloid cells and decrease the production of the proinflammatory cytokine IL-1β [88]. This was due to the fact that *Bmal1* modulates the expression levels of enhancer RNA to regulate time-dependent inflammatory responses following Toll-like receptor 4 (TLR4) expression [89]. The NF-κB family has a profound influence on immunity and inflammation in cancer and promotes oncogenesis. Furthermore, the clock gene CRY has been reported to directly influence inflammatory pathways by activating adenylyl cyclase [90]. Additionally, *Rev-Erbα*, which is another circadian clock gene, enhances the mRNA levels of Cx3cr1 and MMP9 in mouse macrophage by inhibiting the functions of distal enhancers, thereby establishing a macrophage-specific program of repression [91]. Thus, the activation state and modulation of cytokines, chemokines, ROS, miRNAs, and eRNAs in macrophages are directly or indirectly targeted by circadian components. The global or T cell-specific deficiency in BMAL1 does not markedly impact the overall or subset-specific oscillation of T cells [92, 93]. In contrast, mice with a dominant-negative expression of CLOCK, REV-ERBα, or RORα, reportedly have decreased number of Th17 cells in mice [94, 95]. RORγt is essential in the generation, differentiation, and survival of effector subsets of T cells, which possess antitumor properties through the production of the cytokines IL-17A, IL-17F, GM-CSF, and IL-22 and the chemokine CCL20 in mammals [96–99]. Given the importance of Th17 cells in tumor immunity, the circadian clock components in Th17 cells may be promising targets against tumor cells. Although there is increasing research on how circadian clock components affect antigen presentation and T cell priming and activation in tumor immunity, many other aspects need to be explored for further elucidation.

### Circadian clock affects lymphocyte trafficking and infiltration in cancer

The trafficking and homing of lymphocytes play an anti-tumor role under the participation of adhesion chemokines and costimulatory molecules. Except for skin tissue, rhythmic oscillations in the expression of adhesion molecules in other peripheral tissues, including intercellular adhesion molecule (ICAM) 1 (ICAM1) and 2 (ICAM2), and vascular cell adhesion molecule 1 (VCAM1) have been observed. Interestingly, the homing and engraftment of leukemic cells in the leukemia cancer model is strongly time-of-day dependent in mice [100]. Further analysis indicated that the circadian clock gene *Bmal1* regulates the expression of ICAM1 and VCAM1 in endothelial cells or leukocyte subsets, respectively [100]. David et al., reported that the migration of T and B cells strongly exhibited oscillations in a non-continuous manner in lymph nodes, rather than in the thymus and the bone marrow [101]. This phenomenon has been closely linked to the expression of CXCR4, CCL20, and Cx3cr1 and controlled by glucocorticoids, catecholamines, and hypoxia inducible factor 1α (HIF1α) signaling pathway [102, 103]. As observed in mice with deleted *Bmal1*, T cells and B cells ablated oscillations, indicating that cell-autonomous clocks are pivotal for lymphocyte egress and are the critical factors of T and B cell migration [101]. CCL20, which is another cytokine, regulates the homeostatic trafficking of Th17 cells, and are expressed in the small.

intestine according to the time of the day in wild-type mice; the phenomenon was not observed in *Clock*-mutated mice [104]. Chen et al., reported that the core circadian gene *Clock* plays a critical role in the regulation of tumor immunity through the transcriptional upregulation of OLFML3 in glioblastoma, which recruits immune-suppressive microglia into the tumor microenvironment [105]. Collectively, these studies suggest that the subset of lymphocyte trafficking is strongly under the control of cell-intrinsic circadian clock components. Therefore, it is vital to optimize the application of tumor immunotherapy.

As for the infiltration of lymphocytes in the TME, the circadian clock genes in kidney renal clear cell carcinoma, including *Clock*, *Bmal1*, and *Cry1* have been positively correlated with a variety of immune cell infiltrates, such as neutrophil cells, DCs, and CD4+ T cells, respectively [106]. A similar phenomenon was also observed in patients with thoracic cancer. CLOCK and BMAL1 have been closely associated with the infiltration level of CD8+ T cells [107]. Evidence indicates that compared with the expression of BMAL1 in patients with healthy skin, the expression of BMAL1 in patients with melanoma is remarkably disrupted, which represents a dysfunctional circadian clock, and has been positively correlated with the infiltration/activation of T cells [108]. Existing literature indicated that approximately 15% of CD4+ T cells express RORγ in tumor-infiltrating lymphocytes [109]; however, these studies focus on the transcriptional level of clock genes and therefore does not reflect integral changes. The mechanism by which circadian clock modulates tumor immune infiltration remains to be explored.

### Circadian clock components regulate the recognition and elimination of cancer cells

Circadian clock components can modulate cancer antigen-specific T cells and NK cells to kill cancer cells. Immune escape is generally a negative prognostic factor and a predictor of immune checkpoint blockade response in various cancers. Effective immunotherapeutic strategies have been employed this knowledge to treat cancers [110]. Recent researches have expanded our knowledge of the association between the circadian clock and immune escape. In metastatic melanomas, the expression of BMAL1 is responsible for T-cell activation/differentiation markers, and T-cell exhaustion markers, CTLA4, PD-1, and PD-L1 [108]. Further analysis of the functional impact of circadian clock genes indicated that the circadian clock-enriched pathways are enriched in many immune-related pathways, including PD-L1 expression and PD-1 checkpoint pathway in cancer, T cell receptor signaling pathway, and TNF signaling pathway; this corroborates that the circadian clock widely regulates the immunity of tumors [111]. *Per1* and *Cry2* which are two core circadian clock genes, have been linked to the expression of CD4+ T cells, and the expression of PD-1 exhibited a robust circadian rhythm in normal lung tissues [107]. Furthermore, when *Rorγ* is knocked down, the percentage of PD-1^+^ Type 17 cells, along with the levels of PD-1 on individual cells is decreased [109]. These studies suggest that circadian clock genes are potential targets for tumor immunotherapy.

NK cells control the growth and metastasis of tumor cells via an array of activating or inhibitory surface receptors that recognize signs of stressed/pre-malignant cells, including natural killer group 2, member D (NKG2D) and the natural cytotoxicity receptors (NCRs) [112, 113]. Unlike the T cells of adaptive immunity, the activation of NK cells at tumor sites directly destroys malignant cells without prior sensitization. NK cells can secrete a number of cytokines and growth factors, including interferon-γ (IFN-γ), TNF-α, and granulocyte–macrophage colony-stimulating factor (GM-CSF) to kill cancer cells. In rats, chronic shift-lag promotes the growth of lung cancer in rats mainly by altering the circadian rhythm of NK cells, along with cytokines, cytolytic factors, and cytotoxic factors [114]. In a study, when *Per2* or *Bmal1* was knocked down, the secretion of IFN-γ, granzyme B, and perforin in NK cells was significantly decreased [115].

## Clock drugs for the immunotherapy of cancer

The rapid and complementary growth of research on the interaction between the circadian clock and the immunity of cancers is raising new hope for the prevention and treatment of cancer. At present, the application of circadian clock in tumor immunotherapy mainly includes two aspects: drug development for biological clock targets and chrono-immunotherapy.

LYC-53772 and LYC-54143, as potent RORγ synthetic agonists, can boost the differentiation of Th17 cells, block immunosuppression driven by Tregs and significantly elevate the level of secreted cytokines, including IL-17A, IL-17F, and GM-CSF, and IL-22, such that an anti-tumor activity is implicated [116]. In addition, when treated with RORγ agonists, T cells are reportedly resistant to PD-L1 inhibition, which is critical in suppressing antitumor immunity [109]. Moreover, when added during ex vivo expansion, RORγ agonists augment the tumor-eliminating activity of cytotoxic Th17 cells and CAR-T cells, and the cytotoxic activity of human T cells, which can enable the regression of tumors in tumor-bearing mice [109]. Compared with the functions of RORγ agonists-untreated cells, the function of CAR Type 17 cells was elevated when reactivated against a series of tumor cell lines expressing mesothelin and secreted more cytokines, including IL17A and IFNγ [117]. Conssistent with a previous report, when co-infused TRP-1 Th17 and pmel-1 Tc17 cells generated in vitro in the presence of the RORγ agonist, mice with melanoma tumors were protected for more than two months after tumor challenges of over three times, indicating that RORγ agonist-primed cells possessed a stem-like memory phenotype and provided a long-term protection against tumor challenges [117]. Furthermore, the RORα synthetic agonist SR1078 was reported to remarkably increase CD8+ T cell effector responses to anticancer immunity role [118]. Doxorubicin, which is developed from a metabolite of the bacterium Streptomyces peucetius var. Caesius, is one of the commonly employed chemotherapeutic drugs for solid tumors [119]. In a recent study, when mice with Lewis lung carcinoma were treated with doxorubicin, they exhibited a significant alteration in the expression of F4/80 and CD11c in tumor tissues, and in the expression of circadian genes, including *Bmal1*, *Clock*, *Rev-Erbα*, and *Per2* and NF-kB and IL-6 in intraperitoneal macrophages [120].

Experimental immunotherapies indicated that some anticancer treatments are expected to reduce drug toxicity, improve tumor response rate and the duration of the response. As reported, interferon-β (IFN-β), pleiotropic cytokines significant for immune system regulation, exhibited more valid antitumor effect in early light phase of tumor-bearing mice than in early dark phase [121]. In a phase I_II clinical study, IL-2 chronotherapy that was employed to treat metastatic renal cell carcinoma showed moderate toxicity, feasibility in a standard care unit, and activity [122]. These results would be beneficial in applying carefully rationalized medical interventions for the modulation of the circadian clock components, which may be altered in tumors. However, chrono-immunotherapies are still at its initial stage of practice and further investigations of the mechanisms need to be exerted to improve the current anticancer.

## Conclusion

The circadian clock and immunity modulate major process in mammalian physiology and have been heavily studies. Increasing evidence indicates that they can interact with each other through various process in both healthy and pathological states. Tumors and their surrounding can be considered highly complex organs such as, immune cells play an important role in the recognition, death, or maintenance of tumors. Thus, a better understanding of the crosstalk between the circadian clock and cancer-immunity cycle would help in the development of effective immunotherapies for cancer.

## Data Availability

Not applicable.

## Abbreviations

WHO: World Health Organization
SCN: Suprachiasmatic nucleus
ITTFs: Interlocking transcription-translation feedback loops
CLOCK: Circadian locomotor output cycles kaput
Bmal1: Brain and muscle Arnt-like protein-1
PER: Period
CRY: Cryptochrome
NPAS2: Neuronal PAS domain protein 2 (NPAS2)
CK1: Casein kinase 1
NSCLC: Non‑small cell lung cancer
NK: Natural killer
TME: Tumor microenvironment
ECM: Extracellular matrix
NKT: Natural killer T
DCs: Dendritic cells
TAMs: Tumor-associated macrophages
CTLA-4: T-lymphocyte-associated antigen 4
PD-1: Programmed death receptor1
APCs: Antigen-presenting cells
CTLs: Cytotoxic T-lymphocytes
TNF-α: Tumor necrosis factor-α
Th: T helper
PAMPA: Pathogen-associated molecular patterns
TLR4: Toll-like receptor 4
ICAM: Intercellular adhesion molecule
HIF1α: Hypoxia inducible factor 1α
IFN-γ: Interferon-γ
GM-CSF: Granulocyte–macrophage colony-stimulating factor
